# Establishment and Validation of Fourier Transform Infrared Spectroscopy (FT–MIR) Methodology for the Detection of Linoleic Acid in Buffalo Milk

**DOI:** 10.3390/foods12061199

**Published:** 2023-03-12

**Authors:** Zhiqiu Yao, Pei Nie, Xinxin Zhang, Chao Chen, Zhigao An, Ke Wei, Junwei Zhao, Haimiao Lv, Kaifeng Niu, Ying Yang, Wenna Zou, Liguo Yang

**Affiliations:** 1 National Center for International Research on Animal Genetics, Breeding and Reproduction (NCIRAGBR), Ministry of Science and Technology of the People’s Republic of China, Huazhong Agricultural University, Wuhan 430070, China; 2 Key Laboratory of Animal Genetics, Breeding and Reproduction, Ministry of Education, College of Animal Science and Technology, Huazhong Agricultural University, Wuhan 430070, China; 3 College of Veterinary Medicine, Hunan Agricultural University, Changsha 410128, China

**Keywords:** FT-MIR, linoleic acid, machine learning, accuracy profile

## Abstract

Buffalo milk is a dairy product that is considered to have a higher nutritional value compared to cow’s milk. Linoleic acid (LA) is an essential fatty acid that is important for human health. This study aimed to investigate and validate the use of Fourier transform mid-infrared spectroscopy (FT-MIR) for the quantification of the linoleic acid in buffalo milk. Three machine learning models were used to predict linoleic acid content, and random forest was employed to select the most important subset of spectra for improved model performance. The validity of the FT-MIR methods was evaluated in accordance with ICH Q2 (R1) guidelines using the accuracy profile method, and the precision, the accuracy, and the limit of quantification were determined. The results showed that Fourier transform infrared spectroscopy is a suitable technique for the analysis of linoleic acid, with a lower limit of quantification of 0.15 mg/mL milk. Our results showed that FT-MIR spectroscopy is a viable method for LA concentration analysis.

## 1. Introduction

Milk is an important route in humans for nutrient intake, and the fatty acids in milk are associated with many biological functions in humans [1]. The dietary intake of fatty acids has an important influence on coronary disease; specifically, saturated fatty acids (SFA) increase serum cholesterol levels, whereas polyunsaturated fatty acids (PUFAs) reduce the risk of coronary disease [2]. In addition, studies have shown that the fatty acids in milk are also related to the technological properties of milk and the processing of dairy products [3]. The composition of milk fat and fatty acid content reflects to a certain extent the health status of the cow [4].

Linoleic acid (LA) is a type of PUFA that has been shown to have various health benefits, including reducing the risk of chronic diseases and improving insulin sensitivity [5,6]. Milk is an important source of LA, which is considered a potential anticarcinogen and can be manipulated through dietary management [7]. As the economy continues to develop, there is a growing demand for milk that is nutritionally valuable. The dairy industry, therefore, faces two major challenges: (1) aligning the fatty acid composition of milk with consumer preferences, and (2) finding reliable and precise methods to quantify the FA composition of milk [8]. The traditional methods for determining LA content in milk products are gas chromatography (GC) [9] or gas chromatography–mass spectrometry [10], which are time-consuming and labor-intensive and often involve the use of harmful chemicals.

Fourier transform mid-infrared spectrometry (FT-MIR) is a widely utilized analytical technique that has been demonstrated to be effective in a range of applications within the dairy industry. Specifically, FT-MIR has been demonstrated to be valuable in the analysis of antibiotics present in milk [11,12], the quantification of fat and protein content in milk [13], the prediction of methane emissions [14], and the prevention of early lactation diseases in cattle [15,16]. FT-MIR has been increasingly used for the analysis of fatty acids in milk due to its advantages of high throughput in real-time, sensitivity, and low sample preparation requirements [17].

In recent years, there has been a growing interest in using FT-MIR in combination with multivariate analysis techniques, such as partial least squares regression (PLSR), to quantify the PUFA content in milk products. Mid-infrared spectroscopy has been widely used in the rapid prediction of fatty acids. PLSR is probably the most widely used technique in spectral analysis. Many researchers have successfully measured the fat, protein, solid non-fat, and fatty acid content in milk using PLSR regression [18,19,20,21]. With the development of computational power and machine learning methods, more and more multivariate models are used to calibrate the concentration of components in milk. The principal component regression (PCR) algorithm downscales the original features using principal components analysis (PCA) and performs linear regression on the reduced predictor variables, which are the principal components, to predict the target variable. By utilizing a smaller number of principal components that explain the majority of the variance in the data with respect to the target variable, PCR is more effective in mitigating overfitting than linear regression on all original features, particularly for high-dimensional data such as spectra [22]. In recent studies, artificial neural networks (ANNs) have recently been investigated in FT-MIR spectroscopic analysis [23,24]. Random forests (RF) employ an evaluation of the relevance of variables to selectively choose informative variables, thereby facilitating the construction of models that are both parsimonious and robust, and ultimately enhancing the predictive power [25,26].

Some recent endeavors employing FT-MIR spectroscopy have explored quantifying linoleic acid. Beriain et al. [27] predicted the α-linolenic acid and LA in intramuscular fat by using the ANN algorithm and achieved good forecasted results. In the field of dairy analysis, Bonfatti et al. [28] successfully developed a milk fatty acid prediction model for Italian Simmental cattle using MIR with PLSR algorithm on 1040 milk samples. Similarly, Coppa et al. [21] used GC combined with FT-MIR to develop a fatty acid prediction model for 250 Holstein milk samples. However, although GC is a useful technique for analyzing monounsaturated fatty acids, its accuracy may be reduced when analyzing complex mixtures of polyunsaturated fatty acid methyl esters containing trans double bonds, such as LA and alpha-linolenic acid [29]. In addition, the complexity and cost associated with GC analysis for large numbers of samples and the need for expert operators are important factors to consider. Notably, there have been no previous investigations on the prediction of linoleic acid content in buffalo milk using FT-MIR, and only a limited number of studies have assessed the accuracy and precision of FT-MIR-based predictions of PUFA [30]. 

Direct or spectral interference is a common issue in chemical analysis based on spectroscopic methods, where the sensor is not perfectly specific for the analyte [31]. Unintended interference can occur, especially when utilizing PLSR for the compositional analysis of highly complex samples [32]. It is important to carefully evaluate and control potential sources of interference in spectroscopic methods to ensure accurate and reliable chemical analysis. Therefore, the objectives of this study were twofold: (1) to modify the FT-MIR method by incorporating the standard addition technique and establish a more streamlined machine learning prediction model for linoleic acid in milk, and (2) to employ a novel validation strategy to evaluate the accuracy and precision of FT-MIR for the determination of linoleic acid in milk.

## 2. Materials and Methods

### 2.1. Sampling

Over a 3-month period from April to June 2022, milk samples were collected from 31 buffaloes in Hubei, China. For each sampling day, 50 mL of milk was collected in the morning and another 50 mL in the afternoon, and then mixed into a single sample to reflect changes in milk composition throughout the day. In total, 12 L milk samples were collected and stored at −20 °C for further analysis.

### 2.2. FT-MIR and Preprocessing Method

To process the samples, they were first rapidly thawed in a 40 °C water bath and then centrifuged at 2 °C, using a refrigerated centrifuge, at 3000 rpm for 15 min to eliminate fat [33]. The composition of skimmed milk was analyzed using the MilkoScan FT-6000 (FOSS Analytical A/S, Hillerød, Denmark), which revealed that the fat content of whey was less than 0.05%. 

Linoleic acid (LA) was randomly added to skimmed milk samples in seven different concentrations (1, 5, 10, 20, 50, 70, 100 mg/100 mL milk). Isopropanol was utilized as the diluent for the LA [34]. There were 15 samples for each concentration, and a total of 105 samples were used for FT-MIR analysis. MIR spectra were obtained for each sample using the Milkoscan FT 6000. The acquisition was performed twice, and the results were subsequently averaged. The MIR spectra were recorded in the region between 926 and 5012 cm^−1^ and omitted the O–H bending region (1600–1710 cm^−1^) and the O–H stretching region (3020–5012 cm^−1^) due to the high water content in milk [35]. The remaining region (926 to 1618 cm^−1^ and 1705 to 3025 cm^−1^; 524 data points) was selected for analysis [36]. 

**Figure 1:**
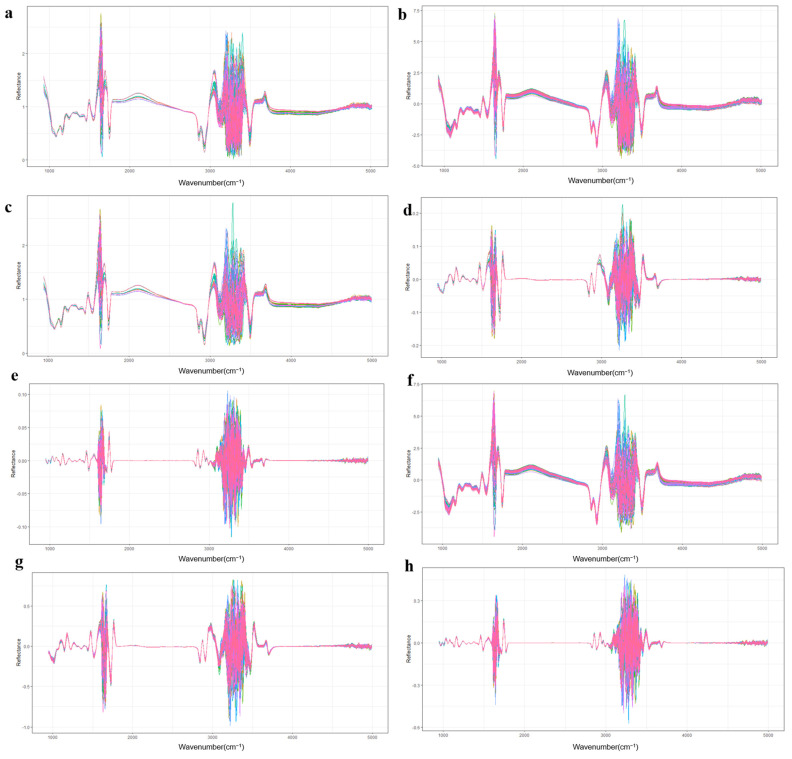
Mid-infrared spectra after various preprocessing methods. (**a**) Raw spectra; (**b**) Mid-infrared spectra after standard normal variables processing; (**c**) Mid-infrared spectra after Savitzky–Golay algorithm processing; (**d**) Mid-infrared spectra after first derivative and Savitzky–Golay algorithm processing; (**e**) Mid-infrared spectra after second derivative and Savitzky–Golay algorithm processing; (**f**) Mid-infrared spectra after SNV and Savitzky–Golay algorithm processing; (**g**) Mid-infrared spectra after SNV, Savitzky–Golay algorithm, and first derivative processing; (**h**) Mid-infrared spectra after SNV, second derivative, and Savitzky–Golay algorithm processing.

To further process the raw spectra, 7 different preprocessing methods were applied, including standard normal variate (SNV), 11-point Savitzky–Golay algorithm (SG), first derivative + Savitzky–Golay algorithm (SG-1), second derivative + Savitzky–Golay algorithm (SG-2), SNV + Savitzky–Golay algorithm (S-SG), SNV + Savitzky–Golay algorithm + first derivative (S-SG-1), and SNV + Savitzky–Golay algorithm + second derivative (S-SG-2) (Figure 1). The R packages “prospect” (version 0.26) and “baseline” (version 1.3-4) were utilized for the preprocessing steps.

### 2.3. Machine Learning Algorithms

In this study, we aimed to determine the optimal quantitative model for the estimation of linoleic acid in milk using various machine learning techniques. All the machine learning algorithms utilized the CARET package version 6.0–93 in R program (version 4.2.2 https://www.r-project.org/ (accessed on 8 September 2022)) [35].

The FT-MIR data (n = 105) was randomly divided into a training set (80%) and a test set (20%) for building and validating the models, respectively. The numerical parameters for each model were entered using the “expand.grid” function and optimized using cross-validation (CV) statistics. We selected the model with the lowest root mean square error of cross-validation (RMSE_CV_) from all preprocessing methods. PLSR is a widely utilized chemometric method in the analysis of spectroscopic data, utilizing latent variables (LV) to decompose the spectral data into systematic variations that account for the observed variance [37]. In comparison, the latent variable of PCR is the number of principal components and the minimum number of principal components required to explain 95% of the variance [38]. ANNs represent a nonlinear extension of traditional linear regression models [39]. While linear regression is limited to modeling linear relationships between features and targets, ANNs have the capability to model complex nonlinear relationships through the utilization of hidden layers [40]. Regularization techniques play a crucial role in preventing overfitting in ANN models, thus improving their accuracy on novel data sets [41]. In the context of the CARET package, the parameter “size” refers to the number of units in a hidden layer, and the parameter “decay” represents the regularization strength. For PLSR and PCR, the maximum number of latent variables was set to 25. The number of the hidden layer for the ANN was varied from 1 to 5, and the decay values were tested for 0, 0.0001, 0.001, 0.01, 0.1, 0.2, 0.3, 0.4, and 0.5. The performance of each model was evaluated using internal 10-fold cross-validation statistics, including RMSE_CV_ and coefficients of determination (R2 cv). The models were then validated by estimating RMSE of prediction (RMSEP) on the external test set.

Random forests (RF) have been demonstrated to hold promise in the realm of feature selection [42]. This procedure involves creating a random forest model and then performing 1000 iterations. Through the creation of a random forest model and subsequent iterations, the importance scores of features were evaluated based on the accuracy of model predictions of the target variable (LA) after replacing the response variable (spectral bands). Spectral bands that are more predictive of the outcome will have relatively high importance scores in each run, while other spectral bands with lower predictivity will only have randomly importance scores. This process enables the significance of features to be calculated [43]. In this study, we employed the rfPermute package (version 2.5.1) in R to perform variable selection using RF. The number of trees utilized in the RF model was set at 500 [44]. The PLS, PCR, and ANN models were again constructed by selecting spectral regions with significance levels less than 0.05. These models underwent variable optimization and performance evaluation in a manner consistent with the methodology previously described.

### 2.4. Quality Control for the Method 

The developed method underwent validation in accordance with the International Conference on Harmonization (ICH) Q2 (R1) guidelines. The Limit of Detection (LOD) was determined by utilizing 10 skimmed milk samples, and calculating the standard deviation of the matrices. The LOD was determined as three times the standard deviation of the ten sample [45].

(1) $$LOD=3\times S_{0}$$

*S*_0_ is the estimated standard deviation of single results at zero concentration.

In terms of relative bias, recovery, repeatability, intermediate precision, lower limit of quantification (LLOQ) and upper limit of quantification (ULOQ), the validation protocol employed a 3 × 5 × 3 (*i* × *k* × *j*) full factorial experiment design [46]. Five different concentration levels (*k*) of linoleic acid (5 mg/100 mL, 10 mg/100 mL, 20 mg/100 mL, 50 mg/100 mL and 100 mg/100 mL) were investigated, with each level being conducted in three replicates (*i*) on three different days (*j*), resulting in a total of 45 samples [46]. 

The trueness of the method was evaluated through the expression of Bias and Recovery.

(2) $$Bias(\%)=\frac{\bar{Y}-Y_{r}}{Y_{r}}\times 100$$

(3) $$Recovery(\%)=\frac{\bar{Y}}{Y_{r}}\times 100$$

where $Y_{r}$ is the theoretical value, $\bar{Y}$ is the average value of a series of measurements.

Precision is evaluated at two levels: repeatability and intermediate precision. This requires the calculation of the mean square of inter-series (MSB) and intra-series (MSE) [47].

If MSE < MSB, then:

(4) $$Repeatability:{\sigma_{Re}}^{2}=MSE$$

(5) $$Intermediate\;precision:{\sigma_{In}}^{2}=\frac{MSB-MSE}{n}$$

Otherwise:

(6) $$Intermediate\;precision=Repeatability=\frac{1}{mn-1}\sum_{i=1}^{m}\sum_{j=1}^{n}{\left(Y_{ij}-\bar{Y}\right)}^{2}$$

where $Y_{ij}$ is the average of the calculated concentration of the *j*-th concentration level of the *i*-th series; *m* is the number of days; *n* is the number of replicates per series.

The current assay acceptance criteria in practice require that at least four out of six samples have an observed mean at the lower limit of quantitation (LLOQ) within 20% of the theoretical value (β = 4/6 ≈ 66.7%), and the observed precision to be ≤20% coefficient of variation [48].

The accuracy profile based on β-content tolerance intervals is a powerful tool for method validation and quality control [49]. This ideal acceptance criterion would ensure that a high proportion (β = 66.7%) of future observations lie within acceptance limits (±20%), with a high level of confidence (confidence level = 0.9). By calculating the lower limit (*L*) and upper limit (*U*) of the tolerance at a particular concentration, the tolerance of the measured value of a specified proportion (β) of all samples will be within the interval [*L*,*U*] with the specified confidence level. It can be considered that if the number of observed values $Y_{n+1}$ within the tolerance interval [*L*,*U*] accounts for more than 0.667 of the total $Y_{n}$, and the confidence level is 0.9, then the detection method is valid, and the formula is shown as follows.

(7) $$Confidence\;level=P\left[P[L\leq Y_{n+1}\geq U/Y_{n}]\geq \beta \right]$$

For instance, for β = 0.667 and confidence level is 0.9, the β- content tolerance interval represents a 90% probability (*p*) that 66.7% of the individual observations of the population are included in the interval [*L*,*U*] [50]. The determination is accepted if the resulting tolerance limits *L* and *U* are completely within acceptance limits (±20%) of the theoretical value; Otherwise, it’s not.

According to Kulkarni’s approach [51], the tolerance interval [*L*,*U*] can be rewritten into the following form:

(8) $$\left[L\left(\%\right),U\left(\%\right)\right]=\left[bias\left(\%\right)-\chi_{k}\times RSD(\%),bias\left(\%\right)+\chi_{k}\times RSD(\%)\right]$$

Where:

(9) $$RSD(\%)=\frac{\sigma_{In}}{Y_{r}}\times 100$$

(10) $$\chi_{k}=\sqrt[{2}]{\frac{k\times \chi_{1;0.667}^{2}(\lambda )}{\chi_{k;0.9}^{2}(0.1)}}$$

$\chi_{1;0.667}^{2}\tau$ is the 66.7th quantile of a noncentral chi-square distribution with the degree of freedom 1. λ is the noncentrality parameter. $\chi_{f^{\prime };0.9}^{2}$(0.1) is the 90th quantile of a noncentral chi-square distribution with the degree of freedom *k*. *χ_k_* denotes the chi-square distribution associated with the variable *k* [50,52].

(11) $$k=\frac{{\left(R^{\prime }+1\right)}^{2}}{{\left(R^{\prime }+\frac{1}{n}\right)}^{2}/\left(m-1\right)+\left(1-\frac{1}{n}\right)/mn}$$

(12) $$\lambda =\frac{nR^{\prime }+1}{mn\left(R^{\prime }+1\right)}$$

(13) $$R^{\prime }=MAX\left[0,\frac{1}{n}\left(\frac{MSB/MSE}{F_{0.85}\left(m(n-1)\right);\left(m-1\right)}-1\right)\right]$$

$F_{0.85}\left(m(n-1)\right);\left(m-1\right)$ is the 85th percentile value of F distribution with the degree of freedom *m*(*n*−1) and *m*−1. The concentration at which the tolerance interval is less than acceptance limits is the limit of quantification. The acceptance limits is typically set at ±20% [52,53].

The procedure for building an accuracy file can be outlined as follows:

(1) Calculate the β-content tolerance interval at a confidence level of 0.9 for each concentration level using Equations (13) or (8), resulting in a lower and upper limit for the interval, denoted as [*L*,*U*].
(2) Graphically represent the results in a 2D plot, with the concentration level plotted on the horizontal axis and the tolerance interval limits (*L*,*U*) plotted on the vertical axis.
(3) Compare the tolerance interval limits (*L*,*U*) with the acceptance limits of −20% to +20% around the theoretical value. If the tolerance interval falls entirely within this acceptance range, the analytical method is deemed valid for the corresponding concentration level. However, if the tolerance interval exceeds these limits, the method is not accepted for use at that concentration level.

## 3. Results and Discussion

### 3.1. Set Up of the Prediction Models

First, we implemented a 10-fold cross-validation process to avoid overfitting. Cross-validation has proven to be a good method for model resampling and is widely used for the mid-infrared prediction of milk composition [54,55]. The performances of the various methods (PLSR, PCR, and ANN) are summarized in Table 1, with the RMSE and coefficient of determination (R^2^). The best model was determined by the smallest RMSE and highest R^2^. The RMSE_CV_ values for the PLSR, PCR, and ANN models were all found to be below ten. In our study, the RMSE_CV_ values of the training set were always lower than the one observed for test set, as mentioned by Soyeurt and Grelet [35]. Results showed that RMSE_CV_ values for PLSR, PCR, and ANN were similar, ranging from 5.1 mg/100 mL–7.3 mg/100 mL, with R^2^_CV_ values also globally similar and ranging from 0.96–0.98. This indicates that the predictive performance of the three models is similar. There were also some differences in correlation values between predictions on the test set. Higher correlation was observed between the predictions given by PLSR and PCR(0.98) compared to those given by ANNs. Our analysis revealed that the PCR method outperformed the PLSR method, with slightly higher predictive accuracy. This difference in performance may be attributed to the distinct component extraction processes employed by PCR and PLSR. Specifically, PLSR identifies regressors from predictors that maximize the covariance with the response variable, while PCR employs principal component analysis (PCA) to identify the direction of greatest variability in the predictor variables and project them into a low-dimensional space to form principal components, which are subsequently used to explain the response variable. The component extraction step in PCR is capable of identifying superior candidate regression components by meticulously scrutinizing the covariance structure among the predictor variables, which may be overlooked by PLSR. Such phenomena have been observed in previous studies as well [56,57]. It is worth noting that the performance of different methods depends on the nature of the analyzed data and the data processing methods used. This is one of the reasons why it is not recommended to use the same milk fatty acid prediction model across different species.

### 3.2. Models Built with the Spectral Regions Selected by RF

Our research on importance measures in random forests has focused on finding data points where the predictor variables are highly correlated. The application of RF results in a significant reduction in the number of variables in each model. The number of data points dropped from 524 before the selection to 135.

**Figure 2:**
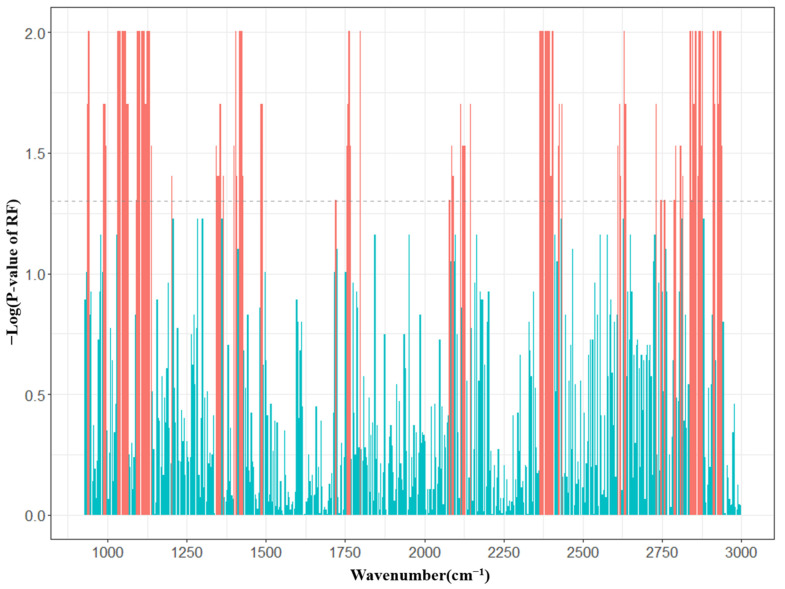
Spectral regions selected by RF for the prediction of linoleic acid. The red domains indicate a significant association with LA (*p* ≤ 0.05), and the green domains indicate a non-significant association with LA (*p* > 0.05).

RF is widely used to assess the importance of features. Wang et al. [58] employed RF feature selection to investigate the relative contributions of soil factors, microbial parameters, and climatic factors in altering soil organic carbon levels. Chen et al. [59] used RF to evaluate the most significant drivers of soil fungal diversity, including plant communities and soil physicochemical properties. Similarly, Andreas et al. [60] utilized RF to identify tillage type as the most important factor affecting dairy cows with Fasciola hepatica, with higher-ranking variables yielding more accurate predictions than those with lower importance scores. The variable importance measure can be used by RF to select and order the spectral regions that are most predictive. Usually, MIR data points are ranked according to decreasing asymptotic *p*-values and importance value. The process of changing random number seeds will result in slightly different results for random forests [61]. Therefore, the response variable was permuted 1000 times to generate new RF models, and the data points that were most correlated with linoleic acid and significant at a *p*-value of less than 0.05 were selected for modeling [62,63]. Most of the selected data points were included in the spectral subsets 940–1215 cm^−1^, 1342–1489 cm^−1^, 2364–2399 cm^−1^, 2823–2935 cm^−1^, and 3715–3846 cm^−1^ (Figure 2). These regions are highly correlated with fatty acids. The first region (940–1215 cm^−1^) is related to the asymmetric vibrations of the C-O-C group in esters; the second region (1342–1489 cm^−1^) is characteristic of the C = O ester Fermi resonance; the third region (1720–1766 cm^−1^) is characteristic of the stretching vibrations of the carbonyl group in esters; the fourth region (2350–2357 cm^−1^) is a synergistic region associated with fatty acids and has been shown to assist in the prediction of fatty acids in milk to some extent [30]. The fourth region (2823–2935 cm^−1^) is characteristic of C-H stretching absorption [64].

The validation results for the prediction models built using the wavelengths selected by RF are presented in Table 2, along with the number of latent variables. The results indicate that compared to the full spectrum model, the RMSE_CV_ value of the RF model is generally lower. The application of RF resulted in a reduction of the R^2^_P_ value by 0.2% in the ANN mode, while the R^2^_P_ value increased by 0.3% in the PCR model. No differences were observed in the R^2^_P_ value of the PLSR model, but the R^2^_cv_ value of PLSR increased by 2.1% after RF feature selection. Thus, the application of RF has produced simpler models, and the predictive power of these simplified models is comparable to that of full spectrum models. As mentioned above, the performance of a method is closely related to the characteristics of the data set, the preprocessing methods, and the relationship between the predictor and response variables. After using RF to extract the original features, the performance of the PLS method was slightly better than that of the PCR method.

In detail, lower RMSE_P_ values were observed between the predictions given by the PLSR and PCR (4.1) compared with ANNs (6.5). This suggests that nonlinear methods, such as ANN models, were not suitable, but linear PLSR showed good performance. Previous research has suggested that FT-MIR predictions with partial least square models are promising approaches [65,66]. This is in agreement with Soyeurt et al. [35], showing that PLSR has better predictive performance than ANNs in orange variety classification. Improving the performance of ANNs requires a large training data set to learn complex data interactions by tuning its hyperparameters, such as size and decay, in this study [67]. In addition, epochs, activation function, and learning rate all affect the predicting capabilities of the ANN. From our results, the ANN does not seem to perform well when the training population is small. The RMSE values for the other four linear models are smaller than those with the ANN, which also suggests that the complex nonlinear relationship between the predictors and target traits is limited [68,69]. It was evident from the values of R^2^ and RMSE that, even though all three models (PLSR, PCR, and SVR) fitted well to the experimental design, PLSR offered better predictive and approximation accuracy. The best predictive performance was achieved by the PLSR with the mean R^2^_P_ value of 0.984 and a RMSEp value of 4.113 mg/100 mL.

We observed a higher predictive ability for linoleic acid content compared with previous studies on FT-MIR predictions, which obtained R^2^ values ranging from 0.43–0.89 [30]. This improvement could be attributed to the expression of fatty acid content estimated in g/100 mL milk, which is more accurate than g/100 g FA [21,70]. Additionally, the utilization of the standard addition method, which is commonly used in the method validation of other analytical methods such as GC, was instrumental in avoiding interference from other fatty acids [34]. To the best of our knowledge, it was the first time that the RF method was used on FT-MIR data to select salient features. Although this method appears promising, further studies will be needed to fully understand its limitations.

### 3.3. MIR Method Validation

The method detection limit for LA was determined using FT-MIR spectroscopy according to the ICH Q2 (R1). The LOD was found to be 3.42 mg/100 mL based on the standard deviation of the blank sample signals (n = 10). The FT-MIR method was validated towards recovery, repeatability, intermediate precision, range, and accuracy for the quantification of LA according to the ICH Q2 (R1). The acceptable limits were set at ±20% for the IR method [53,71]. 

The trueness represents the closeness of the average to the true value, and precision is the closeness among a series of measurements [72]. The uncertainty is a dispersion of measured values from the expected value [73]. The total uncertainty includes the random error and the systematic error. 

Table 3 illustrates that the results for LA at 20, 50, and 100 mg/100 mL concentration levels have good relative bias and recovery, with relative bias ranging from −0.56% to 2.15% and recovery ranging from 99.44% to 102.47%. The repeatability and intermediate precision of LA at 10 mg/100 mL and 20 mg/100 mL concentrations were 1.89% and 2.07% respectively. The repeatability and inter-assay precision of LA at 50 mg/100 mL were 8.34% and 9.46% respectively, while the repeatability and inter-assay precision of LA at 100 mg/100 mL were 11.76% and 13.61%, respectively. In our results, the intermediate precision is worse than the repeatability, which means that there is an effect of day-to-day variability on the spectral data at these concentration levels [74]. 

The accuracy of LA at 20 mg/100 mL (−9.11, 13.41), 50 mg/100 mL (−10.17, 9.07), and 100 mg/100 mL (−10.24, 14.96) concentration were found to be within the acceptable range of −20 to 20%. The accuracy of 5 mg and 10 mg/100 mL level were outside the acceptance limits. This suggests that systematic and random errors increase as the concentration level decreases [46].

**Figure 3:**
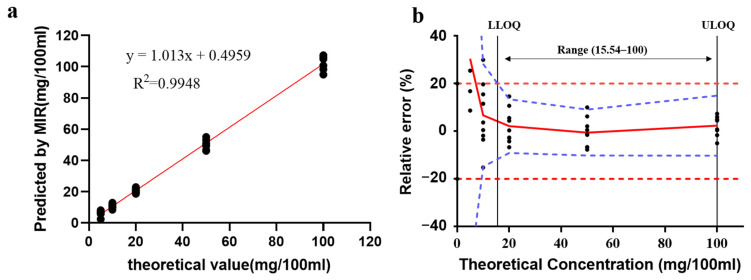
Linear profile and accuracy profile for MIR analysis of the linoleic acid content. (**a**) Correlation graph of MIR predictive values with reference values; (**b**) The red line represents the relative bias, the blue dashed lines are the β-expectation tolerance limits, the red dashed lines are the acceptance limits (±20%), and the 9 black points at each concentration level are relative bias for each predictive value. LLOQ represents the lower limit of quantication, and ULOQ represents the upper limit of quantication.

As shown in Figure 3a, the relationship between the predicted concentrations and the true concentrations was evaluated by the linear equation: y = 1.013x + 0.4959 with R^2^ of 0.9948. The slope and R^2^ values of the linear equation demonstrate the good agreement between the MIR predictions and the theoretical values.

The accuracy profile is a pictorial tool that is widely used for the quality control of medicines [75]. LLOQ and ULOQ are the lowest and highest concentration levels where the β-tolerance expectation limits are included within the acceptable limits. In our study, the LLOQ value was 15.54 mg/100 mL, and the ULOQ value was 100 mg/100 mL. 

In this study the acceptable limit was set at ±20%, and in the other literature the acceptable limit has been set at ±5% to ±30% [50,75,76]. It is a widely recognized standard in the field of bioanalytical methods that pre-study acceptance criteria mandate that the observed mean should be within ±15% of the theoretical value, and the precision’s coefficient of variation should not exceed 15% [50]. The levels of linoleic acid in buffalo milk measured using gas chromatography ranged from 51 mg/100 mL to 85.4 mg/100 mL, which is in between our quantitative ranges [8,77]. Our results show that the MIR method within the quantitative interval fully meets the above criteria.

## 4. Conclusions

The objective of this study was to assess the efficacy of three machine learning models in quantifying the levels of linoleic acid (LA) in raw milk and to theoretically determine the upper and lower bounds of LA quantification. These models included partial least squares (PLSR), principal component regression (PCR), and artificial neural networks (ANNs). The study applied random forest feature selection to the models in order to improve the model performance and reduce complexity. The results of calibration and cross-validation analyses showed that the random forest partial least squares (RF-PLSR) model had the best performance among the three models, with low error values and high regression coefficients. The accuracy profile of the model was further validated using accuracy files, and it was demonstrated that Fourier transform mid-infrared (FT-MIR) could reliably quantify LA levels in the range of 15.54 mg/100 mL to 100 mg/100 mL. In conclusion, the results of this study highlight the potential of FT-MIR as a tool for rapid and reliable identification of LA content in milk. Further research efforts are recommended to develop comprehensive spectral databases for the robust assessment and reliable identification of a wider range of fatty acid concentrations. This will aid in the expansion of FT-MIR in the dairy industry and other relevant fields.

## Figures and Tables

**Table 1:** Performance of 10-fold cross-validation and external validation for predicting LA in milk using 5 different machine learning algorithms ^1^.

	Pre-Processing	LV	RMSE_CV_	RMSE_CV_ SD	R^2^_CV_	RMSEp	R^2^_p_
PLSR	SG-1	nLV ^2^ = 20	7.325	0.546	0.958	4.094	0.984
PCR	SG	Nlv = 17	5.198	0.725	0.980	3.662	0.987
ANN	SNV-SG-1	Size ^3^ = 5 Decay = 0.3	6.274	1.809	0.963	6.426	0.961

^1^. PLSR = partial least squares regression; PCR = principle component regression; ANN = artificial neural network; RMSE_CV_ = root mean square error in cross-validation; R^2^cv = cross-validation R^2^; RMSE_CV_ SD = standard deviation of RMSE_CV_; R^2^_P_ = R2 in prediction; RMSE_P_ = RMSE in prediction. ^2^. nLV = number of latent variables. ^3^. Size = the number of nodes in the hidden layer; decay = the penalty used for ANN.

**Table 2:** Performance of 10-fold cross-validation and external validation of LA in predicted milk based on 5 different machine learning algorithms after random forest algorithm variable selection ^1^.

	Pre-Processing	LV	RMSE_CV_	RMSE_CV_ SD	R^2^_CV_	RMSE_P_	R^2^_P_
PLSR	SG	nLV ^2^ = 8	4.714	1.188	0.983	4.113	0.984
PCR	SG	nLV = 14	5.669	0.836	0.976	4.161	0.983
ANN	SNV	Size ^3^ = 6 Decay = 0.4	7.616	2.373	0.951	6.566	0.959

^1^. PLSR = partial least squares regression; PCR = principle component regression; ANN = artificial neural network. RMSE_CV_ = root mean square error in cross-validation; R^2^cv = cross-validation R^2^; RMSE_CV_ SD = standard deviation of RMSE_CV_; R^2^_P_ = R2 in prediction; RMSE_P_ = RMSE in prediction. ^2^. nLV = number of latent variables. ^3^. Size = the number of nodes in the hidden layer; decay = the penalty used for ANN.

**Table 3:** Trueness, precision, and accuracy results for each concentration level in the validation data ^1^.

		Trueness		Precision		Accuracy	
Level (mg/100 mL)	Mean Calculated Concentration ^2^ (mg/100 mL)	Relative Bias (%)	Recovery (%)	Repeatability (%)	Intermediate Precision (%)	Relative β-Expectation Tolerance Limits (%) ^3^	β-Expectation Tolerance Limits (mg/100 mL) ^4^
5	6.52 ± 1.72	30.4	130.4	3.89	8.21	[−59.18, 119.99]	[2.04, 10.99]
10	10.67 ± 1.29	6.7	106.7	1.89	1.89	[−14.8, 28.21]	[8.51, 12.82]
20	20.43 ± 1.35	2.15	102.15	2.07	2.07	[−9.11, 13.41]	[18.17, 22.68]
50	49.72 ± 2.85	0.56	99.44	8.34	9.46	[−10.17, 9.07]	[44.91, 54.53]
100	102.02 ± 3.42	2.63	102.63	11.76	13.61	[−10.24, 14.96]	[96.05, 108.66]

^1^. The validation criteria are based on the ICH guide to the validation of analytical methods. ^2^. Mean ± SD. ^3^. The β-CTI (%) is the relative β-content tolerance interval. ^4^. Abs β-CTI is the absolute β-content tolerance interval.

## Data Availability

The date are available from the corresponding author.
